# Evaluation of Bone Regeneration in Rat Calvaria Using Bone Autologous Micrografts and Xenografts: Histological and Histomorphometric Analysis

**DOI:** 10.3390/ma13194284

**Published:** 2020-09-25

**Authors:** Carlos R. G. Araújo, Carlo Astarita, Riccardo D’Aquino, André A. Pelegrine

**Affiliations:** 1 Faculdade São Leopoldo Mandic, Instituto São Leopoldo Mandic, Department of Implant Dentistry, Campinas 13045-755, Brazil; carlosrgaraujo@gmail.com (C.R.G.A.); andre.pelegrine@slmandic.edu.br (A.A.P.); 2 Human Brain Wave Srl, corso Galileo Ferraris 63, 10128 Turin, Italy; riccardo.daquino@me.com; 3 Sbarro Institute for Cancer Research and Molecular Medicine, Department of Biology, College of Science and Technology, Temple University, Philadelphia, PA 19122, USA

**Keywords:** regenerative medicine, bone regeneration, autologous tissue

## Abstract

The aim of this study was to investigate the effect of the use of autologous micrografts obtained by the Rigenera® Micrografting Technology and xenograft on critical size defects created in the calvaria of rats. Forty-eight rats were randomly divided into four groups for each of the two evaluation times (15 and 30 days) (*n* = 6). After general anesthesia, a 5-mm diameter bone defect was created in the calvaria of each animal. Each defect was filled with the following materials: blood clot, autologous bone graft, xenograft, and xenograft associated with autologous micrografts. Histomorphometric and histological analysis showed that the group that have received the Rigenera® processed autologous micrografts combined with the xenograft and the group that received autologous bone graft resulted in greater bone formation in both time points when compared with the use of the xenograft alone and blood clot.

## 1. Introduction

Bone defects following trauma have a high impact on the quality of life of millions of people worldwide and innovative regenerative procedures are considered a promising approach to repair these defects. Bone grafting is a surgical procedure aiming to replaces missing bone with natural bone transplants (autografts, allografts, xenografts) or synthetic materials (alloplasts) and is widely used in all fields of oral maxillofacial surgery, especially for implant dentistry [1]. To this end, the use of autogenous bone graft for dental implant installation was first described by Bränemark in 1980, and is currently a well-accepted and widespread procedure in maxillofacial rehabilitation planning [2]. One of the most common challenges in implant dentistry is proper bone reestablishment for implant anchorage in regions that have undergone bone resorption, occasionally, due to missing teeth in the affected region or due to trauma [3]. Bone regeneration of resorbed jaws and pneumatic maxillary sinus is a key factor in successful implant rehabilitation for correct implant placement and appropriate adjustment for future implant-supported prosthesis [4].

A decade ago, a new medical device (Rigeneracons®, Human Brain Wave srl, Torino, Italy) has been introduced into clinical practice. Such a device is able to mechanically disaggregate autologous tissue, samples of a few millimeters in length, which can be taken directly during surgery from the same surgical site. The obtained 80 µm micrografts (i) retain high cellular viability (70–90%), which is the limited step in mechanical disaggregation; (ii) are enriched in progenitor cells; and (iii) more importantly can be used immediately without any manipulation or cell culture [5]. The micrografting technology in successfully applied in oral-maxillofacial surgery, where micrografts derived by human dental pulp or periosteum were able to stimulate periodontal regeneration, atrophic maxillary bone regeneration, alveolar socket preservation and sinus lift augmentation [6,7,8,9]; those applications are recently described in a retrospective review [10].

The application of the Rigenera® technology aim to reduce the morbidity of the donor site making the procedure appealing from the clinical prospective. Based on these considerations, the scope of this study was to evaluate and compare the role of standard autologous bone, xenogenous bone (Bio-Oss®), and micrografts obtained by the Rigenera® technology, for the bone regeneration in critical size defects created in the skull cap of rats. Histological and histomorphometric analyses at two different time points were performed from surgical procedures. The control group was represented by rats where the defects were filled with blood clot.

## 2. Materials and Methods

### 2.1. Animal Model

Forty-eight male Wistar rats weighing between 300 and 400 g and 15 weeks old, from the Bioterismo Center of São Leopoldo Mandic University, were used. The animals were kept in suitable environments with controlled light and temperature, and with ad libitum access to water and feed for laboratory animals. The experiments were carried out in accordance with the Animal Experimentation Ethics Committee (CETEA No. 2018/40).

### 2.2. Experimental Groups

**Figure 1:**
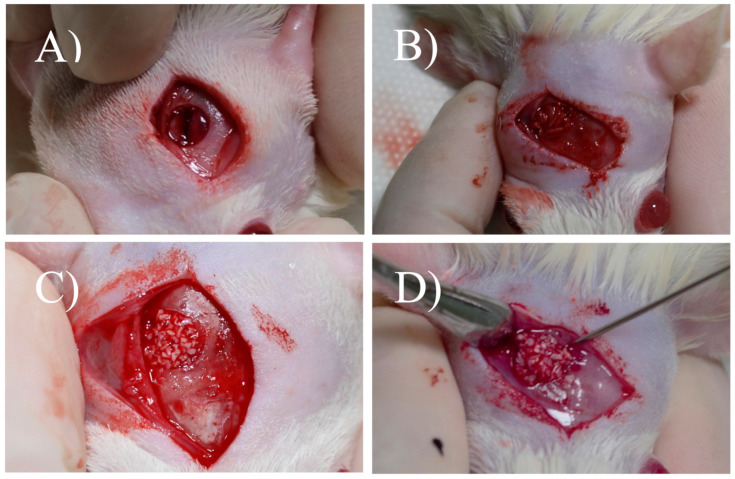
Experimental groups: (**A**) Blood Clot (G1); (**B**) autologous bone (G2); (**C**) xenogenous bone (G3); (**D**) xenogenous bone and Rigenera-obtained micrograft (G4).

The animals were randomly divided into four groups: Group 1 (G1) rats with the bone defect filled with the blood clot; Group 2 (G2) rats with the bone defect filled with autologous bone of the calvaria sample; Group 3 (G3) rats with the defect filled with xenogenous bone (Bio-Oss®); Group 4 (G4) rats with the defect filled with micrografts of calvaria sample plus xenogenous bone (Bio-Oss®). Each group had two different time point, 15 and 30 days from the surgical procedure for the histological and histomorphometric analysis (Figure 1).

### 2.3. Surgical Procedure and Defect Management

The animals were anesthetized by intramuscular injection of xylazine (6 mg/kg) (Rompum®, Bayer, Brazil) and ketamine (70 mg/kg) (Dopalen®, Vetbrands, Brazil). After trichotomy in the frontoparietal region of the animals’ calvaria and antisepsis with 10% polyvinylpyrrolidone (PVPI) with 1% active iodine (Riodeine®, Rioquímica Ltda, São José do Rio Preto, SP, Brazil), the incision was performed with a scalpel blade number 15 (Swann–Morton, Sheffield, England), extending it from the nasofrontal region to the occipital protuberance.

**Figure 2:**
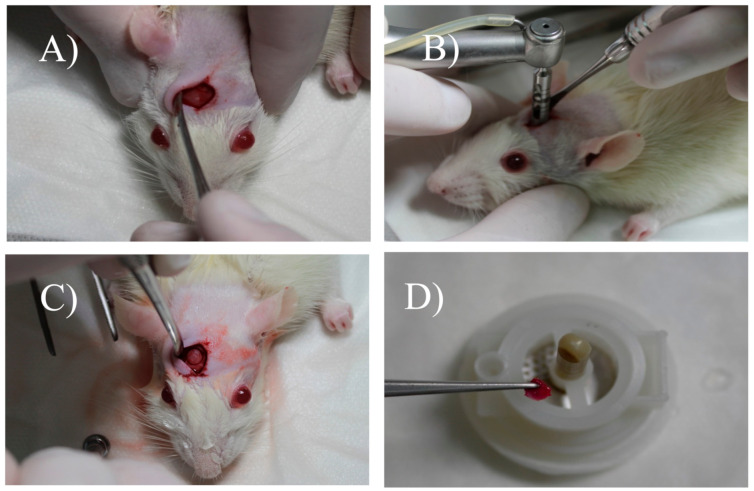
Surgical procedure to create the critical defect in rat calvaria and the use of Rigeneracons. (**A**) Exposure of calvaria; (**B**) use of a 5-mm trephine drill; (**C**) surgical appearance after the creation of the critical defect; (**D**) placement of the bone fragment in the Rigeneracons device.

The skin, the tissues below were detached laterally with a Molt detacher, exposing the calvaria (Figure 2A). With a 5-mm diameter drill bit, coupled to a motor and under abundant refrigeration with sterile saline, a critical size defect was performed in the rat’s calvaria (Figure 2B). After the osteotomy reached the internal cortex of the skull, the bone fragment was removed with the aid of a dentin curette (Hu-Friedy, Chicago, IL, USA) and a 15C scalpel blade (Swann–Morton, Sheffield, England) (Figure 2C). The groups received their due treatments, and in group G1, the defect was filled only with blood clot. Group G2 was treated with autogenous bone removed from the skullcap and particulate. Group G3 had the defect filled with xenograft (Bio-Oss). In Group G4, treated with xenograft associated with micrograft, the autogenous bone collected was also mechanically disaggregated in micrografts by a medical device called Rigeneracons (Human Brain Wave LLC, Turin, Italy) (Figure 2D), without extensive manipulation, safely and easily, as previously reported [5,11,12]. In brief, a sample of bone tissue was collected from the skull of the rat and disgregated using Rigeneracons, with the addition of 0.8 mL of saline solution (0.9% NaCl). The mechanical disintegration was activated with the aid of the contra-angle, inserting the device (80 rpm and 15 Ncm) (Human Brain Wave LLC, Turin, Italy) and after 2 min, the micrograft suspension was collected using of a syringe and placed on the surgical site. The animals were sutured with 5.0 Bioline® resorbable thread.

### 2.4. Painless Induced Death

Forty-eight rats were randomly divided into four groups for each of the two evaluation times (15 and 30 days) (n = 6). The animals from each group were euthanized after 15 and 30 days postoperatively by anesthetic overdose, following the protocol of 90–150 mg/kg of sodium thiopental associated with 10 mg/mL of lidocaine, intraperitoneally. After the animals were euthanized, the skullcap was removed and the parts were fixed in 10% formalina. Then, the pieces were processed for microscopic analysis at the Department of Pathology of São Leopoldo Mandic University, Campinas-SP.

### 2.5. Material Processing

The samples obtained were immersed in a 10% formaldehyde solution immediately after being obtained, where they remained for 24 h. Then, they were subjected to the descaling process by immersion in 20% formic acid. Then, the pieces went through the processes of dehydration and diaphanization in paraffin in order to make semi-serial cuts with a thickness of 5 µm in the longitudinal direction of the piece possible. The cuts obtained were stained with hematoxylin and eosin (HE), and after the assembly of the glass coverslips, they were analyzed under an optical microscope.

### 2.6. Histological and Histomorphometric Analysis

The images were obtained using an optical microscope (LeicaR® DMLB, Heerbrugg, Switzerland) connected to a capture camera (LeicaR® DC 300F Microsystems Ltd., Heerbrugg, Switzerland) and connected to a microcomputer with Axio Vision 4.8 software (Carl Zeiss, Oberkochen, Germany). The scanned images were saved in JPEG format. Histometric evaluation was performed using the Image J 150e software (National Institutes of Health, Maryland, MD, USA), with a 100× magnification. The following criteria based on the work of Melo et al. and Messora et al. [13,14] were used to standardize the histomorphometric analysis of the digitized images.

The total area (TA) analyzed corresponded to the total area of the surgical defect. This area was determined by identifying the internal and external surfaces of the original calvaria on the right and left margins of the surgical defect. These surfaces were connected with lines drawn following their curves. They were then measured from the ends of the specimens 2 mm towards the center of the defect to establish the limits of the original surgical defect. The bone area (BO) was delineated within the limits of the total area (TA). TA was measured in µm^2^ and considered 100% of the area to be analyzed. BO was also measured in µm^2^ and calculated as a percentage of TA.

After standardizing the area of histomorphometry, the following parameters were analyzed:

1. Vital mineralized tissue (VMT), are characterized by the presence of a mineral phase, responsible for rigidity and hardness. VMT represents newly formed bone, so the higher the VMT the level means that we have a quantity of newly formed bone. In histological slides, where the presence of viable cells associated with mineralized tissue was observed, bone tissue was defined as VMT.
2. Non-vital mineralized tissue (NVMT) was defined as xenograft particles (Bio-Oss) defined by polyhedral shapes, without the presence of viable cells and some indicating osseoconduction, High levels of NVMT represent high levels of xenograft remnants.
3. Non-mineralized tissue (NMT) was characterized by granulation tissue and connective tissue, so it can be inferred that high levels of NMT are related to low levels of mineralization.

The Wilcoxon test was used to compare the results obtained, with a *p*-value ≤ 0.05 indicating statistical significance.

## 3. Results

### 3.1. Histological Analysis

**Figure 3:**
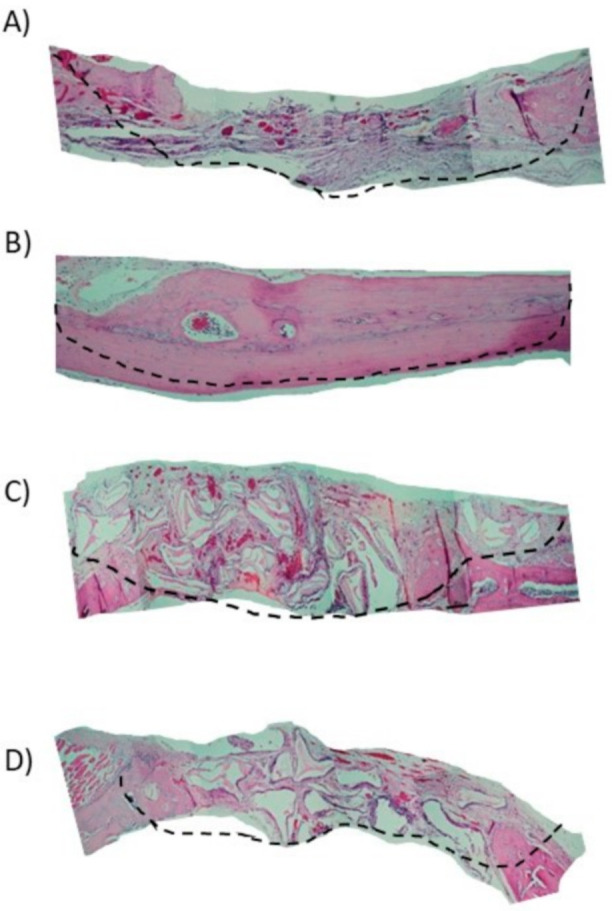
Histological images after 15 days (40× magnification), the line indicates the region of the bone defect (100× magnification)**.** (**A**) Blood clot group (G1); (**B**) autogenous bone group (G2); (**C**) xenogenous bone group (G3); (**D**) xenogenous bone associated with the micro graft group (G4).

**Figure 4:**
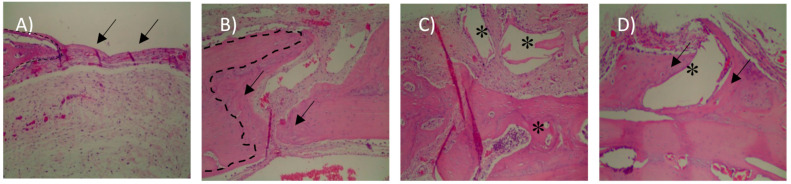
Histological images after 15 days (100× magnification). (**A**) Blood clot group (G1), arrows indicate Osteotomy region and dashed line represent the stump; (**B**) autogenous bone group (G2), observe new bone formation between the stump (dashed line) and the autogenous graft fragment; (**C**) xenogenous bone group (G3)—the asterisks show the particles Bio-Oss®; (**D**) xenogenous bone associated with the micro graft group (G4)—observe the new bone formation around the Bio-Oss® particles.

In the 15-day blood clot group (G1), it was possible to observe the osteotomy line and there was little formation of new bone tissue close to the defect margins (Figure 4A). In most samples of G1 group, the complete defect closure was not observed. The center of the bone defect was generally filled with newly formed bone with a small central area of fibrous tissue rich in fibroblasts and capillaries (Figure 3A).

In the autologous bone group (G2), after 15-days, new bone formation was observed in the regions close to the stumps (Figure 4B). There are different sites of new bone formation with woven and vascularized bone (Figure 3B). In the G3 group where Bio-Oss® was used, the central region of the wound was filled with particles of the biomaterial, which were characterized by negative areas with polyhedral shapes, varied sizes, and contours forming right angles (Figure 3C) and, in some samples, it was possible to observe a newly formed bone in the hole of biomaterial particles that were close to the stump (Figure 4C).

**Figure 5:**
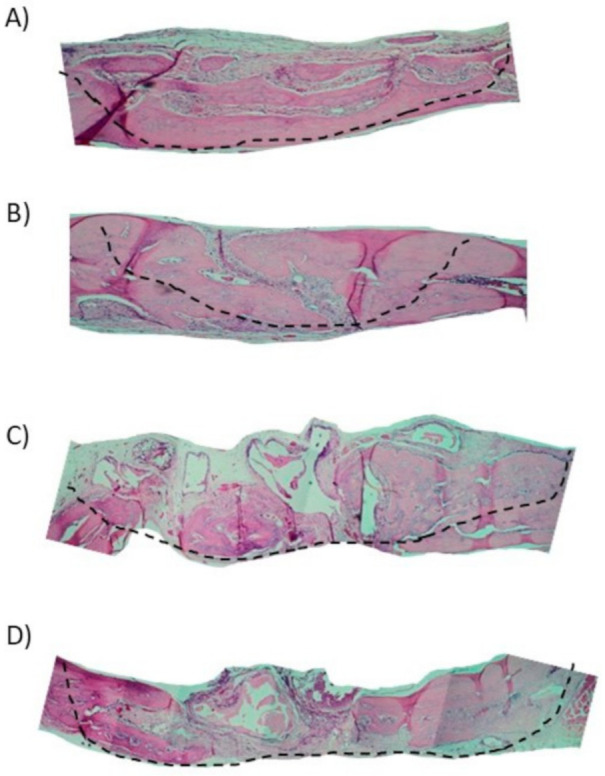
Histological images after 30 days (40× magnification), the line indicates the region of the bone defect (100× magnification). (**A**) Blood clot group (G1); (**B**) autogenous bone group (G2); (**C**) xenogenous bone group (G3); (**D**) xenogenous bone associated with the micro graft group (G4).

In the G4 group composed of Bio-Oss® associated with the micrografts, after 15 days, the presence of Bio-Oss® particles was observed within the defect (Figure 3D), new bone formation was observed from the stump to the center. In the center of the defect, connective tissue rich in fibroblasts, attributable to woven bone, and in some areas, there were regions of new bone formation close to the particles (Figure 5D).

**Figure 6:**
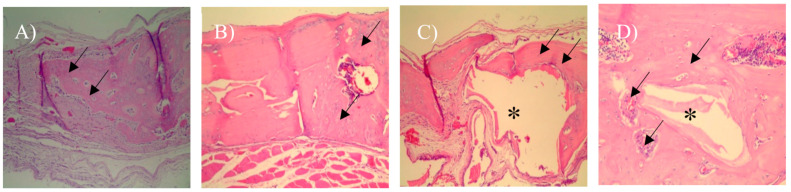
Histological images after 30 days (100× magnification)**.** (**A**) Blood clot group (G1), arrows indicate new bone formation; (**B**) autogenous bone group (G2), observe the new bone formation between the bone particles; (**C**) xenogenous bone group (G3)—the asterisks show the Bio-Oss® particles, observe the new bone formation indicated by the arrows; (**D**) xenogenous bone associated with the micro graft group (G4)—observe the new bone formation around the Bio-Oss® particles and the presence of blood vessels.

With a 100× magnification, it was possible to observe a new bone formation next to the stump in the 30-day blood clot group (G1) (Figure 6A). In the group that the defect was filled with autologous bone (G2), it was possible to observe in some samples a new bone formation filling the entire defect (Figure 6B) and particles of autogenous bone incorporated into the newly formed bone (Figure 5B).

In Group G3 (Figure 5C) it was observed only deproteinized bone particles with different spaces inside filled only with connective tissues. It is possible to observe particles with polyhedral shapes Bio-Oss®, with bone formation around, indicating osteoconduction (Figure 6C).

In the group that received the xenograft associated with the autologous micrograft (Figure 5D), we can observe a bone formation around the Bio-Oss® particle indicating osteoconduction and osteoinduction along with the presence of blood vessels close to the particles which leads the process. (Figure 6D).

### 3.2. Histomorphometric Analysis

**Figure 7:**
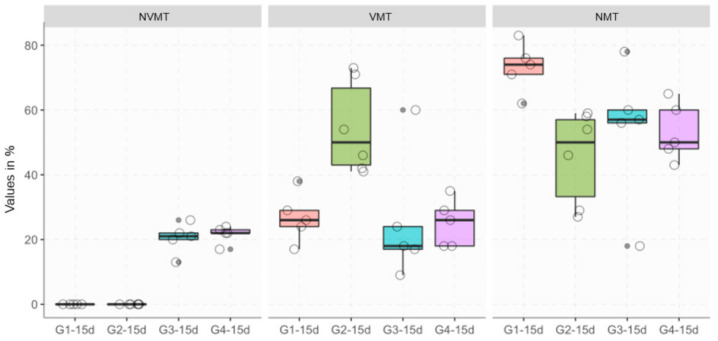
Boxplot of histomorphometric data across the 15-day groups.

**Figure 8:**
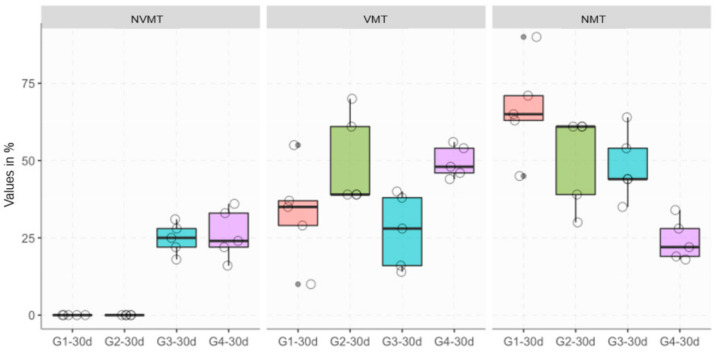
Boxplot of histomorphometric data across the 30-day groups.

The histomorphometric results are shown in Table 1 and Figure 7 and Figure 8. Histomorphometric evaluations showed that there are significant differences in some groups tested according to the parameters analyzed and the healing period. VMT represents newly formed bone, so the higher the VMT the level means that we have a quantity of newly formed bone. NMT represents non-mineralized tissue, so it can be inferred that high levels of NMT are related to low levels of mineralization. High levels of NVMT represent high levels of xenograft remnants (Table 1).

In the results of the vital mineralized tissue (VMT) analyses after 15 days, it can be observed significant differences in all groups (G1, G3, G4) when compared to the group that received autogenous bone (G2) (Figure 7). 

Regarding the presence of non-vital mineralized tissue (NVMT), the analysis showed that there were no statistical differences in the comparison between the groups that received Bio-Oss (G3) when compared with the group that received the Bio-Oss associated with the micrografts (G4) after 15 days, and the variability was absent in groups G1 and G2 (Figure 7).

Regarding non-mineralized tissue (NMT) after 15 days, it was observed significant differences in the clot group (G1) when compared to the group that received autogenous bone (G2) (*p* = 0.0062) and the group that received Bio-Oss with the micrografts (G4) (*p* = 0.0090) (Figure 7).

Concerning the presence of VMT in the analysis after 30 days, the samples that received treatment with autogenous bone (G2) when compared to the test group (G4) showed no differences (*p* = 0.5970), while the analysis of the group with autogenous bone (G2) compared to the group filled only with Bio-Oss® (G3), statistically significant differences were found (*p* = 0.0445). The amount of VMT in the group that received treatment with Bio-Oss® associated with the micrograft (G4) after 30 days when compared to the group in which the defect was filled only with Bio-Oss® (G3) showed a significant difference (*p* = 0.0090).

Regarding the presence of NVMT, the analyzes showed no statistical differences in the comparison between the group (G4) and group (G3). Variability was absent in groups G1 and G2 (Figure 8).

## 4. Discussion

In the present study, bone regeneration capacity of bone micrografts in critical defect repair in rat calvaria associated with xenogenous bone was demonstrated. Stem/progenitor cells can be harvested from various tissues, such as adipose tissue, bone marrow, periosteum, dental pulp, and periodontal ligament [15]. Autologous bone was considered the gold standard for bone regeneration procedures, however, it is often avoided because of the requirement of large samples, with high morbidity of the donor site, since the ratio should be 1:1 with respect to the defect size.

Following the micrografts theory if a graft is disaggregated in small particles it implements better in the recipient site, but this procedure directly reduces the cellular viability imposing a limit in the size achievable. By using the Rigenera® technology, this technical limit is solved since it allows to disaggregate tissue sample in 80 micron-sized micrografts without reducing the cellular viability [5]; taking into account this consideration the amount of tissue harvested is much smaller than the recipient site (not anymore ratio 1:1) and the donor site is the surgical site itself, minimizing the risk of morbidity, allowing a clever use of the bone grafting procedures.

The Rigenera® technology produce within a few minutes a micrografts suspension composed by viable cells surrounded of extracellular matrix (ECM), fragments of ECM and growth factors, opportunely selected by filtration [16]. Moreover, with this tissue micrografts suspension it is possible to soak any kind of scaffolds (i.e., collagen, PLGA, bone substitute) creating in a few minutes a biomaterial, without impacting on significant levels of postoperative morbidity. Previous study confirmed the osteogenic and regenerative properties of the Rigenera® produced micrografts due to the content of the progenitor cells [8]. To date, the Rigenera® micrografting technology has demonstrated its efficacy in different clinical setting. It is widely used with dermal tissue sample for the treatment of complex wounds, such as dehiscences, chronic ulcers, and burns [17,18,19,20,21,22]; for dermatological applications, such as hypertrophic scars [23]; and for the management of androgenetic alopecia [24,25]; with cartilage samples for the management of chondropathy [26,27]; and with bone samples for the treatment of the osteonecrosis of the femoral head [28].

In this study the aim was to challenge the bone regeneration in rats calvaria outcome of autologous bone micrografting plus xenografts compared to the canonical autologous bone grafting and xenografts alone, the filling of the critical defect in rats calvaria performed in this study was done comparing different Groups: G1 blood clot, G2 Autologous bone, G3 xenogenous Bone, and G4 autologous bone micrografts + xenogenous micrografts. The small tissue sample was derived directly from the osteotomy to create the critical defect, so the biological cost was low.

In the present study, histological analysis suggested that the group that received the Rigenera®-obtained micrografts suspension, associated with xenogenous bone, Bio-Oss® (G4) achieved greater bone formation. Statistical analyses have shown that there are significant differences between the groups tested according to the parameters analyzed. The amount of VMT in the group that received the treatment with Bio-Oss® associated with the micrograft (G4) after 30 days when compared with the group where the defect was filled only with Bio-Oss® (G3) showed a statistically significant difference (*p* = 0.0090), suggesting that micrografts were able to promote greater bone formation, corroborating the results obtained in other studies that evaluated bone regeneration with the use of micrografts. However, comparing the samples that received treatment with autogenous bone (G2) and the group that received Bio-Oss® in association with the micrografts (G4) after 30 days, there were no significant differences in the amount of VMT (*p* = 0.5970).

In both groups (G2), where the defect was filled with autogenous bone and (G4), where the defect was filled with autogenous bone micrografts and Bio-Oss® it was possible to observe new bone formation filling the defect (Figure 4B,D), however, only in group G4 we can observe bone formation around the Bio-Oss® along with the presence intense vascularization close to the particles, which leads to a faster regenerative process.

Regarding the euthanasia period, after 15- or 30-days the analysis of the presence of VMT between the groups was studied, and a statistical difference was found in the group that received Bio-Oss in association with the micrograft, suggesting that the studied group accelerated the regeneration process bone (*p* = 0.0088). Regarding the presence of NVMT, the analysis showed that there were no statistical differences in the comparison between the groups, except for those who did not receive Bio-Oss® (i.e., G1 ang G2), suggesting that the amount of material used was similar in both tested groups.

These results provided for the first time on an animal model are in line with previous studies, where different authors reported a reduction in bone resorption and an increase in bone tissue deposition when the Rigenera® autologous micrografts concept was used [15].

## Figures and Tables

**Table 1:** Representation of the descriptive statistics of the analyzed parameters NVMT (Non-vital mineralized tissue), VMT (vital mineralized tissue), NMT (Non mineralized tissue).

Groups	NVMT 15 Days	VMT 15 Days	NMT 15 Days	NVMT 30 Days	VMT 30 Days	NMT 30 Days
**G1**	0.00 ± 0.00	26.80 ± 07.66	73.20 ± 7.66	0.00 ± 0.00	33.20 ± 16.19	66.80 ± 16.19
**G2**	0.00 ± 0.00	54.50 ± 14.32	45.50 ± 14.32	0.00 ± 0.00	49.60 ± 14.86	50.40 ± 14.86
**G3**	20.40 ± 4.72	25.60 ± 19.96	53.80 ± 21.91	24.80 ± 5.07	27.20 ± 12.05	48.20 ± 11.10
**G4**	21.60 ± 2.70	25.20 ± 7.33	53.80 ± 9.04	26.20 ± 8.20	49.60 ± 5.18	24.20 ± 6.72
